# Cost-effectiveness of specialized trauma care: A systematic review

**DOI:** 10.1177/13558196251348409

**Published:** 2025-06-04

**Authors:** Soualio Gnanou, Jason Robert Guertin, Pier-Alexandre Tardif, Blanchard Conombo, Mélanie Bérubé, Natalie Yanchar, Simon Berthelot, Janyce Gnanvi, Lynne Moore

**Affiliations:** 1 Population Health and Optimal Health Practices Research Unit, 4440 Centre de Recherche du CHU de Québec – Université Laval, Québec City, Canada; 2 Department of Social and Preventative Medicine, 4440 Université Laval, Québec City, Canada; 3 Centre de recherche en organogénèse expérimentale de l'Université Laval/LOEX, Québec City, Canada; 4 Faculty of Nursing, 4440 Université Laval, Québec City, Canada; 5 Department of Surgery, 2129 University of Calgary, Calgary, Canada; 6 Department of Family and Emergency Medicine, 4440 Université Laval, Québec City, Canada

**Keywords:** trauma centre, cost-effectiveness, specialized trauma care

## Abstract

**Objectives:**

Several meta-analyses have shown the effectiveness of specialized trauma care, but evidence on cost and cost-effectiveness remains unestablished. We aimed to systematically review evidence on the cost or cost-effectiveness of hospitals specialized in advanced trauma care compared to non or less-specialized hospitals.

**Methods:**

We conducted a systematic review following Preferred Reporting Items for Systematic Reviews and Meta-Analysis (PRISMA) guidelines. We searched PubMed, EMBASE, Cochrane Library, Web-of-Science, EconLit, and grey literature up until June 2024. Two reviewers independently assessed eligibility and extracted relevant data. Reporting quality was assessed using the Consolidated Health Economic Evaluation Reporting Standards (CHEERS) 2022 checklist. Per Cochrane recommendations, findings were synthesized qualitatively.

**Results:**

We identified 4 cost-effectiveness, 3 cost-consequence, and 3 cost-analysis studies, mostly US-based retrospective cohorts. Reporting quality was rated high for 4 studies. All cost-effectiveness studies found specialized trauma centres to be more costly but more effective than non-specialized centres, with incremental cost-effectiveness ratios ranging from 655 to 46,175 Int.$2022 (2022 international dollars) per quality-adjusted life-year (QALY) gained, 43,208 to 999,912 Int.$2022 per life-saved, and 48,567 Int.$2022 per life-year gained. Among cost-consequence studies, two found specialized trauma centres to be costlier and less effective, while one found the opposite. All cost analyses indicated higher costs at specialized trauma centres.

**Conclusions:**

Full economic evaluations identified in this review suggest that specialized trauma care may be cost-effective according to a threshold of $50,000 per QALY. However, our ability to draw conclusions is hampered by the low number of studies, high heterogeneity in study populations and settings, and lack of consideration of trauma systems and of costs beyond the acute phase. Results highlight a critical gap in evidence to guide policymakers in the development of cost-efficient trauma systems.

## Introduction

Injury is one of the leading causes of mortality, disability, and morbidity with over 5 million deaths per year worldwide.^1–4^ Beyond the tragic loss of life and disability, trauma disproportionately affects young people, resulting in a greater loss of productive work years compared to other conditions.^1–3,5^ This results in considerable economic losses for patients, their families, and nations as a whole.^1^ Global costs associated with injury are estimated at US$518 billion.^1^

In response to these challenges, many high-income countries have developed trauma systems, defined as a coordinated network of services and resources for the comprehensive care of trauma patients, covering prevention, emergency care, treatment, and rehabilitation.^6–8^ Within these systems, specialized trauma centres, defined as a hospital providing acute and specialized trauma care, serve as critical hubs for delivering care to severely injured patients according to their level of designation.^9^ Level I trauma centres are highly specialized hospitals providing a full range of services in large urban areas. Level II centres provide similar services but are generally in smaller cities and therefore have lower patient volume. Level III and IV centres play an important role in the trauma system, as they can provide definitive care to patients with minor injuries and initial care to severely injured patients in preparation for transfer to level I or II centres.^6^ These centres each play specific roles and are interconnected through triage, transfer, and coordination protocols that ensure optimal patient care within a trauma system.^6,9^ The implementation of trauma systems has led to substantial reductions in injury-related mortality, disability, and associated costs.^4,10–14^

As health care expenditure continues to rise, decision-makers including policymakers, hospital administrators, and clinicians require evidence on costs and cost-effectiveness to allocate limited resources effectively and to inform strategies that optimize both clinical and economic outcomes. Understanding the economic value of specialized trauma care is therefore crucial for health care decision-making. Cost-effectiveness analysis is a method in health economics that compares the costs and health outcomes of different interventions,^15^ with effectiveness measured using the same units.^16^ Unlike cost-consequence analysis, which presents costs and outcomes separately, or cost analysis, which aggregates the costs of care processes or resources used, cost-effectiveness analysis enables health care providers and policymakers to determine whether the health benefits of an intervention justify its costs.^17^ The commonly used threshold of $50,000 per quality-adjusted life year (QALY) provides a useful benchmark for assessing the cost-effectiveness of interventions, although this figure is open to debate and varies according to context.^16^

Several systematic reviews have synthesized evidence on the effectiveness of specialized trauma care.^12–14,18^ As such, meta-analyses have demonstrated a reduction of 15% in mortality in favor of treatment in a designated trauma centre over a non-trauma centre.^19^ However there is currently no synthesis of data regarding their cost or cost-effectiveness. A systematic review consolidates findings from individual studies, offering an overview of the evidence of the economic value of specialized trauma care. In the context of the unsustainable escalation of health care costs and limited resources, such a synthesis could inform measures to improve the value of trauma care. Our objective was to systematically review evidence of the economic value of acute care in specialized trauma centres compared to non-designated hospitals or those with lower levels of specialization in managing hospitalized patients with acute traumatic injuries.

## Methods

This systematic review was conducted according to Cochrane methodology^20^ and is reported according to Preferred Reporting Items for Systematic Reviews and Meta-Analyses (PRISMA) guidelines (Online Supplement Table S1).^21^ The review protocol was registered with the International Prospective Register of Systematic Reviews (PROSPERO #42023376579) on May 1, 2023. This study was supported by an advisory committee, including experts in trauma care, health economics, and systematic review methodology, who provided guidance throughout the review process.

### Eligibility criteria

We included economic evaluations on patients hospitalized for acute trauma care, including full economic evaluations (cost-effectiveness analysis, cost-utility analysis, and cost-benefit analysis) or partial evaluations (cost-consequence analysis and cost analysis). We considered experimental (randomized and non-randomized clinical trials), observational (prospective cohort, retrospective cohort, and case-control studies), and model-based (simulation) studies comparing acute care hospitals with specialized trauma services (intervention) to either non-designated hospitals or hospitals with lower levels of designation (comparator).

We excluded narrative reviews, research protocols, and conference abstracts. Studies without a comparison group were not considered. Studies exclusively studying military or combat injuries, burns, bites, or late sequelae of injuries were not included.

### Outcomes

We included studies reporting incremental cost-effectiveness ratios (ICERs), incremental cost-utility ratios (ICURs) for full economic evaluations, and added (saved) costs for partial economic evaluations. We considered change in patient health outcomes as measures of effectiveness. All cost measures, regardless of their nature (direct, indirect, or intangible [Online Supplement Table S2]) or currency, were included in our review.

### Data sources and search strategy

We searched MEDLINE (via PubMed), EMBASE, Web of Science, Cochrane Library, and EconLit until June 2024, without any language restrictions. We also searched the grey literature by consulting EThOS, Trove - National Library of Australia, and ProQuest Dissertations & Theses Global.

A search strategy was developed for PubMed using Boolean operators, relevant keywords, and terms related to “trauma centre”, “injury”, “cost-effectiveness” and “cost analysis”. We then adapted this strategy to the other databases (Online Supplement Table S3). We consulted an experienced librarian (FB), as well as experts in economic evaluation (JRG, BC), trauma systems (LM), and a specialist in systematic review and meta-analysis (PAT) to refine our search strategy using the Peer Review of Electronic Search Strategies (PRESS) checklist.^22^ We assessed sensitivity by checking whether our search strategy identified four sentinel studies, identified a priori.^23–26^

### Selection process

We piloted the selection process on three samples of 500 studies until acceptable agreement was reached based on the team’s extensive experience with systematic reviews including reviews of economic evaluations.^27,28^ After the pilot phase, pairs of independent reviewers (SG, BC, PAT, LM) screened titles and abstracts of retrieved studies. The same pairs of reviewers then assessed potentially eligible full texts against inclusion criteria. Disagreements between reviewers were resolved through discussion to reach a consensus. The opinion of other reviewers with expertise in economic evaluation (JRG) and trauma systems (LM) was sought to settle any remaining discrepancies.

We used EndNote 20 software (Version 20.5 (Bld 16,860)) for reference management. Duplicate records were identified and removed through electronic and manual screening processes. In cases where multiple publications were based on the same data, we included the most recent study.

### Data items and abstraction process

An electronic data extraction form with detailed instructions was created and piloted on a representative sample of 10 studies. Pairs of reviewers with methodological and content expertise (SG, PAT, LM) independently extracted the following information from eligible articles: study design (experimental, observational, simulation-based), type of economic evaluation (cost-effectiveness, cost-utility, cost-consequence, cost analysis), population (age, injury type, injury severity), setting (country, year, hospital designation), the perspective of the economic evaluation (patient, hospital/clinic, health system, societal), time horizon, discount rate, intervention, comparator and outcome. Where information regarding study eligibility was ambiguous or missing, we attempted to obtain the information by emailing the first, second, and last listed authors up to three times.

### Data synthesis

The study selection process was depicted using a PRISMA flow diagram. As per Cochrane recommendations for systematic reviews of economic evaluations, we did not conduct meta-analyses due to variability in cost estimates across different settings and studies.^20^ The results were summarized qualitatively. In addition to costs reported in the original studies (Online Supplement Table S4), we presented costs standardized to 2022 international dollars^29^ (Int.$2022) to facilitate comparisons between studies.

### Reporting quality of included studies

Two content experts (SG, BC) independently assessed reporting quality using the 28-item Consolidated Health Economic Evaluation Reporting Standards 2022 (CHEERS) 2022 checklist.^30^ A third senior research team member was consulted in the case of disagreement (JRG). This guidance applies to all types of health economic evaluations, encompassing cost analyses and cost-consequence analyses.^30^ Non applicable items were excluded, and overall compliance was assessed by calculating the proportion of satisfied criteria among those that were applicable. Specifically, items 11 to 13 were considered non-applicable for cost analyses, and items 16 and 22 for studies that were not simulation-based. Articles meeting over 75%, 50%–75%, and under 50% of applicable criteria were deemed to have high, moderate, and low-quality reporting, respectively.^31^

### Subgroup analyses

We planned qualitative subgroup analyses by age (pediatric < 19 years; adult 20-64 years; geriatric ≥ 65 years), type of injury (traumatic brain injury; spinal cord injury; solid organ injury; orthopedic injury; multisystem injury), injury severity (minor ISS < 12; major ISS ≥ 12), country, type of economic evaluation (cost-effectiveness; cost-utility; cost-consequence and cost-analysis), year of study, World Bank country classification (low-middle income; high-income), type of study (experimental; observational; model-based), perspective (Ministry of Health; Societal; Hospital; Patient), reporting quality (low; moderate; high).

## Results

### Results of the search

**Figure 1.**
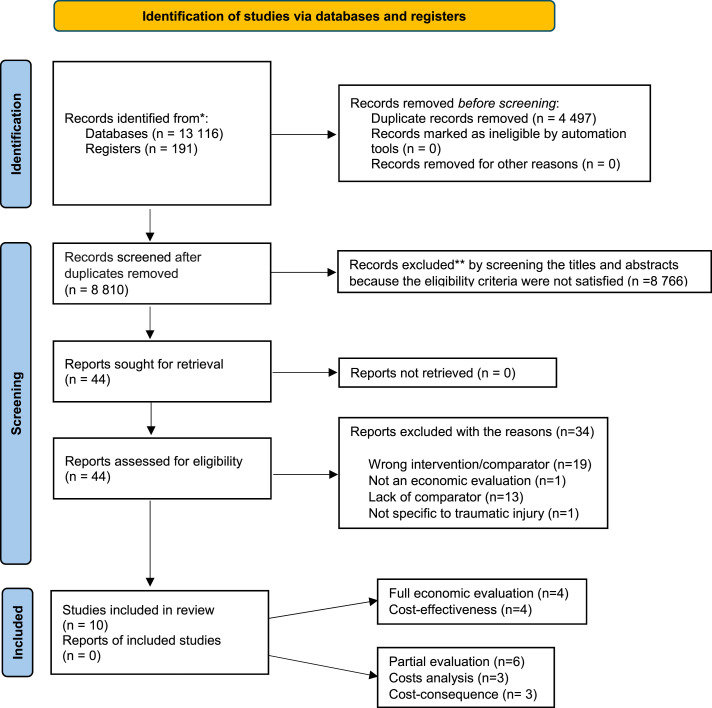
PRISMA 2020 flow diagram for new systematic reviews which included searches of databases and registers only. *Consider, if feasible to do so, reporting the number of records identified from each database or register searched (rather than the total number across all databases/registers). If automation tools were used, indicate how many records were excluded by a human and how many were excluded by automation tools. From: Page MJ, McKenzie JE, Bossuyt PM, Boutron I, Hoffmann TC, Mulrow CD, et al. The PRISMA 2020 statement: an updated guideline for reporting systematic reviews. BMJ 2021;372:n71. doi: 10.1136/bmj.n7.

A total of 10 studies^23–26,32–37^ were considered eligible and were included in the review (Figure 1). Among these, four evaluated cost-effectiveness,^23–26^ three assessed cost-consequences,^32,36,37^ and three were cost-analysis studies.^33–35^

### Characteristics of included studies

All included studies were from high-income countries (Table 1). The majority were conducted in the United States^23,24,32–34,36,37^ (*n* = 7), followed by Canada^26,35^ (*n* = 2) and the United Kingdom (*n* = 1).^25^ Nine studies were retrospective cohorts, and one was prospective.^24^ Four studies reported full economic evaluations^23–26^ and six reported partial evaluations.^33–35^ Nine studies used direct costs and one both direct and indirect costs.^24^ The interventions assessed included trauma system,^25^ trauma centres,^23,24,26,33–35,37^ trauma services,^36^ and trauma units.^32^ The comparators were jurisdictions without a formal trauma system,^25^ non-trauma centres,^23,24,26,33–35,37^ non-trauma services,^36^ non-trauma units,^32^ and less-specialized (Level II, III, or IV) trauma centres.^33–35^

**Table 1.** Characteristics of included studies.

First author Publication year Country	Study design	Economic evaluation	Injury population; Setting	Perspective	Time horizon; Discount rate	Intervention(s)	Comparator(s)	Outcome(s)
O’Kelly 1990 UK	Retrospective	Cost-effectiveness	Major trauma (ISS > 12); UK National Health Service	Ministry of Health	NR	Trauma system proposed by the RCSE, ‘ideal’ trauma system	No trauma system, No trauma system	ICER ($ per QALY)
Goldfarb 1996 USA	Retrospective	Cost-analysis	Major trauma (ISS > 13); 104 hospitals in the USA	NR	NR	Level I trauma centre Level I trauma centre Level II trauma centre	Non-trauma centre Level II trauma centre Non-trauma centre	Costs
Séguin 1999 Canada	Retrospective	Cost-effectiveness	Major trauma (ISS > 12); Ottawa general hospital	Hospital	1-year (3% for survival)	Tertiary trauma centre	Non-trauma centre	ICER ($ per QALY)
Durham 2006 USA	Retrospective	Cost-effectiveness	All trauma; Acute care hospitals in Florida	NR	NR	Trauma centres	Non-trauma centres	ICER (marginal cost per life saved, marginal charge per life saved)
MacKenzie 2010 USA	Prospective	Cost-effectiveness	Moderate and major trauma (AIS ≥ 3); 69 hospitals in 14 USA states.	Societal	1-year and lifetime. (3% for lifetime)	Level I trauma centre	Non-trauma centre	ICER ($ per life year gained, life saved, QALY)
Zocchi 2015 USA	Retrospective	Cost-consequence	Minor and moderate trauma (ISS < 15); Acute care hospitals in California	NR	NR	Trauma centres	Non-trauma centres	Costs mortality
Mabry 2015 USA	Retrospective	Cost-analysis	Minor trauma (ISS < 9) and major trauma (ISS ≥ 9); Trauma centres in Arkansas	NR	NR	Level I and II trauma centres	Level III and IV trauma centres	Costs
Porgo 2019 Canada	Retrospective	Cost-analysis	Moderate and major trauma (death, ICU admission, LOS > 2 days, transfer-in); Trauma centre in Québec, Canada	NR	NR	Level I trauma centres	Level II, III, IV trauma centres	Costs
Scott 2020 USA	Retrospective	Cost-consequence	Minor and moderate trauma (AIS ≤ 3); Level 1 trauma centre in New Jersey	NR	NR	Trauma services	No trauma services	Costs complications
Bauman 2022 USA	Retrospective	Cost- consequence	Trauma admissions with no ICU stay; Level I, trauma centre in Nebraska	NR	NR	Trauma units	Non-trauma units	Costs complications

Abbreviations: UK, United Kingdom; ISS, Injury Severity Score; NR, Not Reported; RCSE, Royal College of Surgeons of England; ICER, Incremental Cost-Effectiveness Ratio; QALY, Quality-Adjusted Life Years; USA, United States of America; AIS, Abbreviated Injury Scale; ICU, Intensive Care Unit; LOS, Length Of Stay.

### Reporting quality

Among the 10 studies examined, four had high reporting quality, that is they reported more than 75% of the CHEERS guide^30^ items,^24,26,35,37^ while the other six had moderate reporting quality, that is between 50 and 75% of items^23,25,26,32–34,36^ (Table 2). More than 50% of the included studies did not report key items such as the title, perspective, time horizon, discount rate, currency, distributional effects, the effect of uncertainty, source of funding, and conflicts of interest.

**Table 2.** Consolidated health economic evaluation of reporting with standards 2022 (CHEERS 2022).

First author publication year	Title	Abstract	Background	Analysis plan	Population	Setting	Comparator	Perspectives	Time horizon	Discount rate	Health issues	Effectiveness	Outcomes	Resources	Currency	Model choice	Assumptions	Heterogeneity	Distributional effects	Characterizing uncertainty	Approach to engagement	Parameters	Main results	Effect of uncertainty	Effect of engagement	Discussion	Funding	Conflicts	% Items	Reporting quality
Cost-effectiveness																														
MacKenzie 2010^1^	×	✓	✓	✓	✓	✓	✓	✓	✓	✓	✓	✓	✓	✓	✓	NA	✓	✓	✓	✓	NA	NA	✓	✓	NA	✓	✓	×	92	High
Durham 2006^2^	×	✓	✓	✓	✓	✓	✓	×	×	×	✓	✓	✓	✓	✓	NA	✓	✓	✓	✓	NA	NA	✓	×	NA	✓	×	×	71	Moderate
Séguin 1999^3^	✓	✓	✓	✓	✓	✓	✓	✓	✓	✓	✓	✓	✓	✓	×	✓	✓	✓	×	×	NA	✓	✓	×	NA	✓	×	×	77	High
O'Kelly 1990^4^	×	✓	✓	✓	✓	✓	✓	✓	×	×	✓	✓	✓	✓	×	NA	✓	×	×	×	NA	NA	✓	×	NA	✓	×	×	58	Moderate
Cost consequence																														
Bauman 2022^5^	×	✓	✓	NA	✓	✓	✓	×	×	×	✓	✓	✓	✓	×	✓	✓	✓	×	×	NA	✓	✓	×	NA	✓	×	✓	64	Moderate
Scott 2020^6^	×	✓	✓	NA	✓	✓	✓	×	×	×	✓	✓	✓	✓	×	NA	✓	✓	×	×	NA	NA	✓	×	NA	✓	✓	✓	65	Moderate
Zocchi 2015^7^	✓	✓	✓	NA	✓	✓	✓	×	×	×	✓	✓	✓	✓	×	✓	✓	✓	✓	✓	NA	✓	✓	×	NA	✓	✓	✓	80	High
Cost analysis																														
Porgo 2019^8^	×	✓	✓	NA	✓	✓	✓	×	×	×	NA	NA	NA	✓	✓	✓	✓	✓	✓	✓	NA	✓	✓	✓	NA	✓	✓	×	77	High
Mabry 2015^9^	×	✓	✓	NA	✓	✓	✓	×	×	×	NA	NA	NA	✓	✓	NA	×	✓	×	×	NA	NA	✓	×	NA	✓	✓	✓	64	Moderate
Goldfarb 19961^10^	×	✓	✓	NA	✓	✓	✓	×	×	×	NA	NA	NA	✓	×	✓	✓	✓	×	✓	NA	✓	✓	✓	NA	×	×	×	59	Moderate
% Item	**20**	**100**	**100**	**100**	**100**	**100**	**100**	**30**	**20**	**20**	100	**100**	**100**	**100**	**40**	**100**	**90**	**90**	**40**	**50**	**0**	**100**	**100**	**30**	**0**	**90**	**40**	**30**		

✓: Information provided.

×: Information not provided.

NA: Not Applicable.

### Economic evaluation findings

#### Full economic evaluations

In the four full economic evaluations, specialized trauma centres were associated with higher costs and higher effectiveness than non or less specialized centres^23–26^ (Table 3). ICERs, presented in 2022 international dollars (Int.$2022) per unit of effectiveness, varied between $655 to $46,175 per QALY,^24–26^ $43,208 to $999,912 per life saved,^23,24^ and $48,567 per life year gained.^24^

**Table 3.** Economic evaluation with the conversion of costs into 2022 international dollars.

First author Publication year	Intervention; Comparator	Costs included	Cost, Int$2022^a^			Effectiveness			Outcome measure^b^	Cost or cost-effectiveness
			Measure	Intervention	Com parator	Measure	Intervention	Comparator	Value	
Cost-effectiveness										
MacKenzie 2010^1^	Level I trauma centre; Non-trauma centre	Direct and indirect costs	Adjusted mean total costs per patient (lifetime) Adjusted mean total costs per patient (lifetime) Adjusted mean total costs per patient (one year)	280 676 280 676 99 754	246 679 246 679 72 511	Mean life-years gained per patient Net lives saved (per 100 admissions) QALY	18.5 0.896 0.59^c^	17.8 0.862 -	$48 567 per life-year gained $999 912 per life saved $46 175 per QALY	More costly and more effective
Durham 2006^2^	Trauma centres; Non-trauma centres	Direct costs	Mean costs per patient Mean charge per patient	15 689 76 498	7 998 47 461	Differential survival Differential survival	0.178^c^ 0.178^c^	- -	$43 208 per life saved $163 129 per life saved	More costly and more effective
Séguin 1999^3^	Tertiary trauma centre; Non-trauma centre	Direct costs	Mean costs per patient	18 662	12 441	Mean discounted QALY	8.3	6.91	$4476 per QALY	More costly and more effective
O'Kelly 1990^4^	Trauma system proposed by the RCSE,‘Ideal’ trauma system; No trauma system	Direct costs	Costs per centre, per year	23 972 018^d^ 11 196 807^d^	- -	QALYs gained per centre per year QALYs gained per centre per year	9 029^c^ 2 887^c^	- -	$3878 per QALY $655 per QALY	More costly and more effective
Cost consequence										
Bauman 2022^5^	Trauma units: Non-trauma units	Direct costs	Adjusted direct costs	4941	5639	Complications (%)	7.8	13.5	NA	Less costly and more effective
Scott 2020^6^	Trauma services involved in care; Trauma services not involved in care	Direct costs	Median costs per patient (propensity-matched)| Median charges per patient (propensity-matched)	17 307 81 605	12 586 54 327	Complications (%) Complications (%)	5 5	1 1	NA NA	More costly and less effective
Zocchi 2015^7^	Trauma centre; Non-trauma centre	Direct costs	Mean costs per patient	17 282	12 956	Mortality rate (per 1000 hospitalizations)	6.9	5.7	NA	More costly and less effective
Costs analysis										
Porgo 2019^8^	Level 1 trauma centre; Level II trauma centre, level III trauma centre, level IV trauma centre	Direct costs	Median costs per patient	5185 5185 5185	4243 4031 3459	NA NA NA	NA NA NA	NA NA NA	NA NA NA	More costly
Mabry 2015^9^	Level I and II trauma centres; Level III and IV trauma centres	Direct costs	Median costs per patient	15 637 15 637	9756 9254	NA NA NA	NA NA NA	NA NA NA	NA NA NA	More costly
Goldfarb 1996^10^	Level I trauma centre; Level II trauma centre Non-trauma centre	Direct costs	Adjusted mean charges per patient	20 182 20 182	7 609 13 450	NA NA NA	NA NA NA	NA NA NA	NA NA NA	More costly

QALY, Quality-Adjusted Life Years.

Ideal’ trauma system: a trauma system that includes all the components identified with optimal trauma care, such as prevention, access, prehospital care and transportation, acute hospital care, rehabilitation, and research activities.

NA: Not Applicable.

^a^All prices were converted to 2022 international dollars (CCEMG - EPPI-Centre Cost Converter).

^b^Outcomes are reported with the conversion of costs.

^c^Only differential effectiveness was calculated.

^d^Only differential costs were calculated.

#### Partial economic evaluations

Findings from the cost and cost-consequence analysis studies were mixed (Table 3). In two of the three cost-consequence studies^36,37^ specialized trauma centres were associated with higher costs ($17,307 vs $12,586, $81,605 vs $54,327 and $17,282 vs $12,956 (Int.$2022) per patient), and lower effectiveness (5% vs 1% for complications and 6.9 vs 5.7 per 1000 hospitalizations for mortality) compared to non-specialized or less specialized centres. The third showed the opposite ($4941 vs $5639 and 7.8% vs 13.5% for complications).^32^ In the three cost-analysis studies^33–35^ specialized trauma care was associated with higher costs than less specialized care; costs ranged from $3459 to $20,182 (Int.$2022) per patient.

#### Subgroup analyses

Costs per QALY in full economic evaluations from the study from the United States^24^ were 10 times higher than the 2 studies from countries with universal health care (Canada and the United Kingdom).^25,26^ Two^24,26^ out of three^24,26,37^ high-quality reports found specialized trauma centres to be more effective than non-specialized hospitals and all found them to be more costly. We were unable to conduct subgroup analyses for age and injury severity because only one study evaluated cost-effectiveness in these subgroups. This study reported that trauma centres were more cost-effective for patients under 55 years of age than those aged 55 and over and more cost-effective in severely injured patients than those with less severe injuries. We were also unable to conduct subgroup analyses for injury type, type of economic evaluation, World Bank income country classification, type of study and perspective due to lack of studies.

## Discussion

In this systematic review, we identified 10 studies evaluating the costs or cost-effectiveness of specialized trauma care. However, only 4 were full economic evaluations, less than half had high reporting quality, heterogeneity in study populations and settings was high, and only one study considered costs beyond acute care or conducted subgroup analyses. Despite these significant limitations, our results suggest that specialized trauma care may have the potential to be cost-effective. Specifically, all cost-effectiveness studies suggested that specialized trauma centres are more costly, albeit more effective.^23–26^ The three cost-utility^24–26^ studies all reported costs per QALY of less than $50,000, suggesting that specialized trauma care is cost-effective at this commonly used threshold.^38^ Findings from the three cost-consequence studies were mixed. One study found specialized trauma care to be less costly and more effective,^32^ while two studies found it to be more costly and less effective.^36,37^ However, these studies were all conducted in populations with minor to moderate trauma. In the three-cost analysis studies^33–35^ specialized trauma care was more costly.

We did not identify any systematic reviews synthesizing evidence on cost or cost-effectiveness of specialized care in other health care domains. However, our findings are in line with a cost-effectiveness analysis comparing specialized multidisciplinary clinics for the management of heart failure in Ontario, Canada with standard hospitals, conducted from a Ministry of Health perspective.^39^ The results indicated that these clinics were more costly but also more effective, with an ICER of 18,259 CAD. However, our results diverge from an economic analysis conducted in Germany from a health system perspective, revealing that colon cancer-certified oncology centres were more effective and less costly than their noncertified counterparts.^40^ According to the authors, the higher costs in noncertified oncology centres could be attributed to confounding whereby patients in these centres had more preexisting medical conditions and potentially required additional resources, thereby contributing to higher overall costs. An economic evaluation comparing Geriatric Fracture Centres to usual care centres from a societal perspective revealed that specialized centres had lower costs but were less effective than non-specialized centres.^41^ Authors hypothesized that lower costs could be explained by shorter lengths of stay in specialized centres whereas lower effectiveness in terms of quality of life may be explained by residual confounding, whereby patients treated in specialized centres had more complex health conditions. These discrepancies highlight the need to consider the specificities of each area of health care setting in interpreting the results of economic evaluations.

### Limitations of the body of evidence

First, most studies did not consider indirect costs or costs / effectiveness beyond the acute care phase. This likely led to an underestimation of the benefit of specialized trauma care over non-specialized care, given the major long-term consequences of trauma in terms of loss of productivity and expenses related to rehabilitation and the treatment of complications.^42,43^ Overlooking these expenses could lead to a significant underestimation of real health care costs and to information bias in comparisons if the contributions of this cost item differed across groups. Second, we observed significant heterogeneity in study populations. Full economic evaluations included major trauma, i.e., patients likely to most benefit from specialized care, whereas cost-consequence studies were based on minor to moderate trauma, i.e., patients that do not require highly specialized care. This may explain why these studies mostly observed lower effectiveness and higher costs in specialized trauma centres.^33,34^ In line with this, only one study conducted subgroup analyses,^24^ limiting our ability to identify the subgroups of patients for whom specialized trauma care may be more cost-effective. Third, the body of evidence does not consider trauma centres in the context of trauma systems. This approach is key to taking account of the fact that a trauma system is designed for centres to play specific, inter-dependent roles within the system. Fourth, the assessment of included studies with the 2022 CHEERS guideline^30^ revealed some limitations in the reporting quality of included studies. Few studies adequately detailed perspectives, time horizons, discount rates, assumptions, and parameters of the economic analysis. This lack of clarity hinders interpretability, reproducibility, and generalizability of the results, compromising the robustness of study conclusions. Furthermore, the retrospective nature of most studies exposes them to selection biases (e.g., representativeness of the general population), information bias (e.g., incomplete, or inaccurate data) and most importantly, indication bias (i.e., sicker patients are more likely to be transferred to a trauma centre). Fifth, studies included in this review were conducted in only three countries (USA, Canada, and UK) and most were conducted before 2015, which limits the generalizability of results to current trauma care outside these jurisdictions.

### Limitations of the review process

Although this study was conducted according to rigorous methodology, certain limitations should be noted. Firstly, the intrinsic methodological quality of the included studies was not formally assessed as available tools cannot be applied across different types of economic evaluations, limiting comparability. Instead, we evaluated reporting quality using the CHEERS 2022 tool. However, reporting quality has been found to be highly correlated to methodological quality in economic evaluations.^28^ Secondly, many elements of the CHEERS tool did not apply to cost and cost-consequence studies. Finally, despite an exhaustive search strategy, some relevant studies may have been missed. Economic evaluations are often only conducted when interventions prove cost-effective,^28^ which could potentially lead to a publication bias that may overestimate the cost-effectiveness of specialized trauma centres. The results obtained should therefore be interpreted with caution.

### Implications for practice, policy, and research

The gaps in evidence identified in this review underline questions that need to be answered in future research to inform trauma system development. For example, how do long-term health consequences and costs affect cost-effectiveness? Which patients could be treated in less specialized or non-specialized centres, for example moderately complex orthopedic injuries or elderly patients with mild complicated traumatic brain injury? What is the most cost-effective configuration of trauma systems in terms of the number of centres, their geographical disposition, and levels of designation? How does the type of payment model influence cost-effectiveness? To answer these questions, we need to conduct robust full economic evaluations using real-world acute care and follow-up data, across diverse health care settings and populations.

## Conclusion

Full economic evaluations identified in this review suggested that specialized trauma centres may have the potential to be cost-effective at a threshold of 50,000 International $ per QALY. However, our ability to draw definitive conclusions about the economic value of specialized trauma centres is hampered by the low number of studies, high heterogeneity in study populations and settings, and lack of consideration of costs beyond the acute care phase. High-quality economic evaluations are urgently needed to inform policymakers on the optimal organization of specialized trauma care.

## Supplemental Material

Supplemental Material - Cost-effectiveness of specialized trauma care: A systematic review Supplemental Material for Cost-effectiveness of specialized trauma care: A systematic review by Soualio Gnanou, Jason Robert Guertin, Pier-Alexandre Tardif, Blanchard Conombo, Mélanie Bérubé, Natalie Yanchar, Simon Berthelot, Janyce Gnanvi and Lynne Moore in Journal of Health Services Research & Policy
