# Supplementary material for: Cost-effectiveness of specialized trauma care: A systematic review
Source: J Health Serv Res Policy. 2025 Jun 4;31(1):56–67. doi: 10.1177/13558196251348409 (PMC12647394; doi:10.1177/13558196251348409)
Supplement: Supplemental Material - Cost-effectiveness of specialized trauma care: A systematic review [file sj-pdf-1-hsr-10.1177_13558196251348409.pdf]

ONLINE SUPPLEMENT

Online Supplement Table S1. PRISMA Checklist

Online Supplement Table S2. Cost items considered in included studies

Online Supplement Table S3. Electronic Search Strategies

Online Supplement Table S4. Results of economic evaluation without cost conversion

Online Supplement. Glossary of terms for economic evaluations

Online Supplement Table S1. PRISMA 2020 Checklist

Section and Topic	Item #	Checklist item	Location where item is reported
**TITLE**			
Title	1	Identify the report as a systematic review.	page 1
**ABSTRACT**			
Abstract	2	See the PRISMA 2020 for Abstracts checklist.	page 1,2
**INTRODUCTION**			
Rationale	3	Describe the rationale for the review in the context of existing knowledge.	page 3,4
Objectives	4	Provide an explicit statement of the objective(s) or question(s) the review addresses.	page 4
**METHODS**			
Eligibility criteria	5	Specify the inclusion and exclusion criteria for the review and how studies were grouped for the syntheses.	page 4,5
Information sources	6	Specify all databases, registers, websites, organisations, reference lists and other sources searched or consulted to identify studies. Specify the date when each source was last searched or consulted.	page 5,6
Search strategy	7	Present the full search strategies for all databases, registers and websites, including any filters and limits used.	page 5,6
Selection process	8	Specify the methods used to decide whether a study met the inclusion criteria of the review, including how many reviewers screened each record and each report retrieved, whether they worked independently, and if applicable, details of automation tools used in the process.	page 6
Data collection process	9	Specify the methods used to collect data from reports, including how many reviewers collected data from each report, whether they worked independently, any processes for obtaining or confirming data from study investigators, and if applicable, details of automation tools used in the process.	page 6,7
Data items	10a	List and define all outcomes for which data were sought. Specify whether all results that were compatible with each outcome domain in each study were sought (e.g. for all measures, time points, analyses), and if not, the methods used to decide which results to collect.	page 5
	10b	List and define all other variables for which data were sought (e.g. participant and intervention characteristics, funding sources). Describe any assumptions made about any missing or unclear information.	page 6,7
Study risk of bias assessment	11	Specify the methods used to assess risk of bias in the included studies, including details of the tool(s) used, how many reviewers assessed each study and whether they worked independently, and if applicable, details of automation tools used in the process.	
Effect measures	12	Specify for each outcome the effect measure(s) (e.g. risk ratio, mean difference) used in the synthesis or presentation of results.	
Synthesis methods	13a	Describe the processes used to decide which studies were eligible for each synthesis (e.g. tabulating the study intervention characteristics and comparing against the planned groups for each synthesis (item #5)).	page 7
	13b	Describe any methods required to prepare the data for presentation or synthesis, such as	page 7

Section and Topic	Item #	Checklist item	Location where item is reported
		handling of missing summary statistics, or data conversions.	
	13c	Describe any methods used to tabulate or visually display results of individual studies and syntheses.	page 7
	13d	Describe any methods used to synthesize results and provide a rationale for the choice(s). If meta-analysis was performed, describe the model(s), method(s) to identify the presence and extent of statistical heterogeneity, and software package(s) used.	page 7
	13e	Describe any methods used to explore possible causes of heterogeneity among study results (e.g. subgroup analysis, meta-regression).	page 7,8
	13f	Describe any sensitivity analyses conducted to assess robustness of the synthesized results.	
Reporting bias assessment	14	Describe any methods used to assess risk of bias due to missing results in a synthesis (arising from reporting biases).	
Certainty assessment	15	Describe any methods used to assess certainty (or confidence) in the body of evidence for an outcome.	
**RESULTS**			
Study selection	16a	Describe the results of the search and selection process, from the number of records identified in the search to the number of studies included in the review, ideally using a flow diagram.	page 8
	16b	Cite studies that might appear to meet the inclusion criteria, but which were excluded, and explain why they were excluded.	page 8
Study characteristics	17	Cite each included study and present its characteristics.	page 8
Risk of bias in studies	18	Present assessments of risk of bias for each included study.	
Results of individual studies	19	For all outcomes, present, for each study: (a) summary statistics for each group (where appropriate) and (b) an effect estimate and its precision (e.g. confidence/credible interval), ideally using structured tables or plots.	page 9
Results of syntheses	20a	For each synthesis, briefly summarise the characteristics and risk of bias among contributing studies.	
	20b	Present results of all statistical syntheses conducted. If meta-analysis was done, present for each the summary estimate and its precision (e.g. confidence/credible interval) and measures of statistical heterogeneity. If comparing groups, describe the direction of the effect.	page 9
	20c	Present results of all investigations of possible causes of heterogeneity among study results.	page 9,10
	20d	Present results of all sensitivity analyses conducted to assess the robustness of the synthesized results.	
Reporting biases	21	Present assessments of risk of bias due to missing results (arising from reporting biases) for each synthesis assessed.	
Certainty of evidence	22	Present assessments of certainty (or confidence) in the body of evidence for each outcome assessed.	
**DISCUSSION**			
Discussion	23a	Provide a general interpretation of the results in the context of other evidence.	page 10-11
	23b	Discuss any limitations of the evidence included in the review.	page 11-

Section and Topic	Item #	Checklist item	Location where item is reported
			12
	23c	Discuss any limitations of the review processes used.	page 12
	23d	Discuss implications of the results for practice, policy, and future research.	page 12,13
**OTHER INFORMATION**			
Registration and protocol	24a	Provide registration information for the review, including register name and registration number, or state that the review was not registered.	page 4
	24b	Indicate where the review protocol can be accessed, or state that a protocol was not prepared.	page 4
	24c	Describe and explain any amendments to information provided at registration or in the protocol.	
Support	25	Describe sources of financial or non-financial support for the review, and the role of the funders or sponsors in the review.	Title page
Competing interests	26	Declare any competing interests of review authors.	Title page
Availability of data, code and other materials	27	Report which of the following are publicly available and where they can be found: template data collection forms; data extracted from included studies; data used for all analyses; analytic code; any other materials used in the review.	

Online Supplement Table S2. Cost items considered included in studies

Study	Direct costs	Indirect costs
MacKenzie[1]	Index hospitalization<br>Pre-index hospital care<br>Post-index hospitalization inpatient care<br>Rehospitalizations for acute care<br>Rehabilitation<br>Long-term care<br>Post-index hospitalization outpatient care<br>Physical/occupational therapy<br>Hospital transport costs<br>Other outpatient services<br>Home health	Informal care from family and friends
Durham[2]	Hospital Costs	-
Séguin[3]	Imaging<br>Laboratory<br>Rehabilitation and social work<br>Respiratory therapy<br>Operating room<br>Pharmaceuticals<br>Nursing	-
O'Kelly[4]	Hospitalization cost<br>Consultant<br>Theatre operating costs<br>Capital costs	-
Bauman[5]	Hospital Costs<br>Hospital length of stay	-
Scott[6]	Hospital Costs<br>Hospital charges	-
Zocchi[7]	Hospitalization cost	-
Porgo[8]	Operating room<br>Medical Imaging (X-ray radiography, ultrasound)<br>Magnetic resonance imaging (head, face, neck, spinal cord, thorax, abdomen, pelvis, rachis, full body, other)<br>Computed tomography (head, face, neck, thorax, abdomen, pelvis, rachis, full body, other)<br>Angiography<br>Emergency department<br>Intensive care unit<br>Medical ward<br>Paraclinical services (Physiotherapy, Occupational therapy, Psychotherapy, Respiratory therapy)	-
Mabry[9]	Medicare Cost<br>Hospital length of stay	-
Goldfarb[10]	Hospital charges<br>Hospital length of stay	-

Online Supplement Table S3. Search strategy for PubMed (30-06-2024)

Research	***Concepts***	***PubMed search strategy***	***# Results***
**#1**	Trauma centre<br>(Controlled vocabulary)	"Advanced Trauma Life Support Care"[Mesh] OR "Trauma Centres"[Mesh] OR "Trauma Nursing"[Mesh] OR "Emergency Medical Services"[Mesh]	172,496
**#2**	Trauma centre<br>(Free text)	"trauma cent*"[TIAB] OR "trauma unit*"[TIAB] OR "trauma system*"[TIAB] OR "trauma department*"[TIAB] OR "trauma organization*"[TIAB] OR "trauma organisation*"[TIAB] OR "Trauma servic*"[TIAB] OR "Emergency Medical Service*"[TIAB] OR "emergency health care servic*"[TIAB] OR emergency accident service*[TIAB] OR "emergency centre*"[TIAB] OR emergency dispensary*[TIAB] OR "emergency medical service*"[TIAB]	39,732
**#3**	Total Trauma centre	#1 OR #2	191,680
**#4**	Injuries<br>(controlled vocabulary)	"Wounds and Injuries"[Mesh]	1,025,189
**#5**	Injuries<br>(Free text)	Fractur*[TIAB] OR Injur*[TIAB] OR trauma*[TIAB] OR "Wound*"[TIAB]	1,751,435
**#6**	Total Injuries	#4 OR #5	2,174,760
**#7**	Cost-effectiveness or costs (economic evaluation)<br>(Controlled vocabulary)	"Cost-effectiveness analysis"[Mesh] OR costs [Mesh] OR "Cost Benefit Analysis"[Mesh] OR "Health Care Costs"[Mesh]	268,579
**#8**	Cost-effectiveness or costs (economic evaluation)<br>(Free text)	economic*[TIAB] OR "marginal analys*"[TIAB] OR "Benefit and Cost*"[TIAB] OR "Costs and Cost Analysis*"[TIAB] OR "Cost benefit*"[TIAB] OR "Health Care Costs*"[TIAB] OR "Cost consequence*"[TIAB] OR "Cost effective*"[TIAB] OR "Cost utility*"[TIAB] OR "Direct Service Costs"[TIAB] OR "Employer Health Costs"[TIAB] OR "Hospital Costs"[TIAB] OR Costs*[TIAB]	768,853
**#9**	Total Cost-effectiveness or costs (economic evaluation)	#7 OR #8	898,091
**#10**	Final	#3 AND #6 AND #9	3,595

Online Supplement Table S3. Search strategy for Embase (30-06-2024)

Research	***Concepts***	***Embase search strategy***	***# Results***
#1	Trauma centre<br>(controlled vocabulary)	'emergency health service'/exp OR 'advanced trauma life support care'/exp	339,778
#2	Trauma centre<br>(Free text)	'trauma cent*':ti,ab,kw OR 'trauma unit*':ti,ab,kw OR 'trauma system*':ti,ab,kw OR 'trauma department*':ti,ab,kw OR 'trauma organization*':ti,ab,kw OR 'trauma service*':ti,ab,kw OR 'emergency health care servic*':ti,ab,kw OR 'emergency accident service*':ti,ab,kw OR 'emergency centre*':ti,ab,kw OR 'emergency dispensary*':ti,ab,kw OR 'emergency medical service*':ti,ab,kw	53,684
#3	Total Trauma centre	#1 OR #2	359,070
#4	Injuries<br>(controlled vocabulary)	'injury'/exp	2,876,222
#5	Injuries<br>(Free text)	fractur*:ab,ti,kw OR injur*:ab,ti,kw OR trauma*:ab,ti,kw OR wound*:ab,ti,kw	2281,449
#6	Total Injuries	#4 OR #5	3,690,321
#7	Cost-effectiveness or costs<br>(economic analysis)<br>(controlled vocabulary)	'cost-effectiveness analysis'/exp OR 'Cost Benefit Analysis'/exp *OR* 'Health Care Costs'/exp OR Costs/exp	778,499
#8	Cost-effectiveness or costs<br>(economic analysis)<br>(Free text)	economic*:ab,ti OR 'marginal analys*':ab,ti OR 'cost effectiveness*':ab,ti OR 'cost effectiveness ratio*':ab,ti OR 'cost efficiency analysis*':ab,ti OR 'cost benefit analysis*':ab,ti OR 'benefit and costs*':ab,ti OR 'health care costs*':ab,ti OR 'cost and cost analysis*':ab,ti OR 'cost benefit*':ab,ti OR 'cost consequence*':ab,ti OR 'cost effective*':ab,ti OR 'cost utility*':ab,ti OR 'direct service costs*':ab,ti OR 'employer health costs*':ab,ti OR 'hospital costs*':ab,ti OR 'Costs*':ab,ti	964,078
#9	Total Cost effectiveness or costs<br>(economic analysis)	#7 OR #8	1,247,101
#10	Final	#3 AND #6 AND #9	6,502

Online Supplement Table S3. Search strategy for Cochrane (30-06-2024)

Research	***Concepts***	***Cochrane search strategy***	***# Results***
**#1**		MeSH descriptor: [Emergency Medical Service] explode all trees	4,704
**#2**		MeSH descriptor: [Trauma Centres] explode all trees	2,038
**#3**		MeSH descriptor: [Advanced Trauma Life Support Care] explode all trees	445
**#4**	Trauma centre (controlled vocabulary)	#1 OR #2 OR #3	6,701
**#5**	Trauma centre (Free text)	'trauma cent*':ti,ab,kw OR 'trauma unit*':ti,ab,kw OR 'trauma system*':ti,ab,kw OR 'trauma department*':ti,ab,kw OR 'trauma organization*':ti,ab,kw OR 'trauma service*':ti,ab,kw OR 'emergency health care servic*':ti,ab,kw OR 'emergency accident service*':ti,ab,kw OR 'emergency centre*':ti,ab,kw OR 'emergency dispensary*':ti,ab,kw OR 'emergency medical service*':ti,ab,kw	22,661
**#6**	Total Trauma centre	#4 OR #5	23,405
**#7**	Injuries (controlled vocabulary)	MeSH descriptor: [Wounds and Injuries] explode all trees	5,772
**#8**	Injuries (Free text)	fractur*:ab,ti,kw OR injur*:ab,ti,kw OR trauma*:ab,ti,kw OR wound*:ab,ti,kw	149,773
**#9**	Total Injuries	#7 OR #8	149,816
**#10**		MeSH descriptor: [Cost-Benefit Analysis] explode all trees	14,139
**#11**		MeSH descriptor: [Health Care Costs] explode all trees	21,942
**#12**		MeSH descriptor: [Costs] explode all trees	41,324
**#13**	Cost effectiveness or costs (economic analysis***)*** (controlled vocabulary)	#10 OR #11 0R #12	14,145
**#14**	Cost effectiveness or costs (economic analysis***)*** (Free text)	economic*:ab,ti OR 'marginal analys*':ab,ti OR 'cost effectiveness*':ab,ti OR 'cost effectiveness ratio*':ab,ti OR 'cost efficiency analysis*':ab,ti OR 'cost benefit analysis*':ab,ti OR 'benefit and costs*':ab,ti OR 'health care costs*':ab,ti OR 'cost and cost analysis*':ab,ti OR 'cost benefit*':ab,ti OR 'cost consequence*':ab,ti OR 'cost effective*':ab,ti OR 'cost utility*':ab,ti OR 'direct service costs*':ab,ti OR 'employer health costs*':ab,ti OR 'hospital costs*':ab,ti OR 'Costs':ab,ti	91,446
**#15**	Total cost effectiveness or costs (economic analysis***)***	#13 OR #14	92,325
**#16**	Final	#6 AND #9 AND #14	1,874

Online Supplement Table S3. Search strategy for Web of Science (30-06-2024)

Research	***Concepts***	***Web of science search strategy***	***# Results***
#1	Trauma centre (Free text)	TS=("trauma cent*" OR "trauma unit*" OR "trauma system*" OR "trauma department*" OR "trauma organization*" OR "trauma service*" OR "emergency health care servic*" OR "emergency accident service*" OR "emergency centre*" OR "emergency dispensary*" OR "emergency medical service*")	41,365
#2	Injuries (Free text)	TS=(fractur* OR injur* OR trauma* OR wound*)	2,405,101
#3	Cost-effectiveness or costs (economic analysis) (Free text)	TS=(economic* OR "marginal analys*" OR "cost effectiveness*" OR "cost effectiveness ratio*" OR "cost efficiency analysis*" OR "cost benefit analysis*" OR "benefit and costs*" OR "health care costs*" OR "cost and cost analysis*" OR "cost benefit*" OR "cost consequence*" OR "cost effective*" OR "cost utility*" OR "direct service costs*" OR "employer health costs*" OR "hospital costs*" OR "Costs**")**	2,610,005
#4	Final	#1 AND #2 AND #3	1,823

Online Supplement Table S3. Search strategy for EconLit (30-06-2024)

Research	***Concepts***	***EconLit Search Strategy***	***# Results***
**S1**		noft(Emergency Medical Services)	245
**S2**		noft(Trauma Centres)	47
**S3**	Trauma centre<br>(controlled vocabulary)	S1 OR S2	289
**S4**	Trauma centre<br>(Free text)	ab,ti,kw**(**trauma cent* OR trauma unit* OR trauma system* OR trauma department* OR trauma organization* OR trauma service* OR emergency health care servic* OR emergency accident service* OR emergency centre* OR emergency dispensary* OR emergency medical service*)	***567***
**S5**	Total Trauma centre	S3 OR S4	612
**S6**	Injuries<br>(controlled vocabulary)	noft(wounds AND injuries)	8
**S7**	Injuries<br>(Free text)	ab,ti,kw(fractur* OR injur* OR trauma* OR wound*)	3,829
**S8**	Total Injuries	S6 OR S7	3,831
**S9**		noft(Cost-Benefit Analysis)	9,593
**S10**		noft(Health Care Costs)	10,922
**S11**		noft(Costs)	220,247
**S12**	Cost effectiveness (economic analysis***)***<br>(controlled vocabulary)	S9 OR S10 OR S11	220,247
**S13**	Cost effectiveness (economic analysis***)***<br>(Free text)	ab,ti(economic* OR marginal analys* OR cost effectiveness* OR cost effectiveness ratio* OR cost efficiency analysis* OR cost benefit analysis* OR benefit and costs* OR health care costs* OR cost and cost analysis* OR cost benefit* OR cost consequence* OR cost effective* OR cost utility* OR direct service costs* OR employer health costs* OR hospital costs* OR costs*)	455,738
**S14**	Total Cost effectiveness or costs<br>(economic analysis***)***	S12 OR 13	536,954
**S15**	Final	S5 AND S8 AND S14	53

Online Supplement Table S4. Results of economic evaluation without cost conversion

First author<br>Publication year	Intervention;<br>Comparator	Costs included	Costs			Effectiveness			Outcome measure‡	Cost or cost-effectiveness
			**Measure**	**Intervention**	**Comparator**	**Measure**	**Intervention**	**Comparator**	**Value**	
**Cost-effectiveness**										
MacKenzie<br>2010[1]	Level I trauma centre;<br>Non-trauma centre	Direct and indirect costs	Adjusted mean total costs per patient (Lifetime)<br>Adjusted mean total costs per patient (Lifetime)<br>Adjusted mean total costs per patient (One year)	225 747<br>225 747<br>80 232	198 403<br>198 403<br>58 320	Mean life-years gained per patient<br>Net lives saved (per 100 admissions)<br>QALY	18.5<br>89.6<br>0.59†	17.8<br>86.2<br>-	$36 319 per life-year gained<br>$790 931 per life saved<br>$36 961 per QALY	More costly and more effective
Durham<br>2006[2]	Trauma centres;<br>Non-trauma centres	Direct cost	Mean cost per patient<br>Mean charge per patient	11 825<br>57 657	6 028<br>35 772	Differential survival<br>Differential survival	0.178†<br>0.178†	-<br>-	$32 514 per life saved<br>$122,75 per life saved	More costly and more effective
Séguin<br>1999[3]	Tertiary trauma centre;<br>Non-trauma centre	Direct cost	Mean cost per patient	14 115	9 410	Mean discounted QALY	8.3	6.91	$4 303 per QALY	More costly and more effective
O'Kelly<br>1990[4]	Trauma system proposed by the RCSE,<br>'Ideal' trauma system;<br>No trauma system	Direct cost	Cost per centre per year	8 505 000*<br>3 972 500*	-<br>-	QALYs gained per centre per year<br>QALYs gained per centre per year	9 029†<br>2 887†	-<br>-	$1 376 per QALY<br>$942 per QALY	More costly and more effective
**Cost consequence**										
Bauman<br>20225	Trauma units;<br>Non-trauma units	Direct cost	Direct costs	4 941	5 639	Complications (%)	7.8	13.5	NA	Less costly and more effective
Scott<br>2020[6]	Trauma services involved in care;<br>Trauma services not involved in care	Direct cost	Median cost per patient (Propensity-matched)<br>Median charges per patient (Propensity-matched)	16 611<br>78 323	12 080<br>52 142	Complications (%)<br>Complications (%)	5<br>5	1<br>1	NA<br>NA	More costly and less effective
Zocchi<br>2015[7]	Trauma centre;<br>Non-trauma centre	Direct cost	Mean costs per patient.	15 155	11 361	Mortality rate (per 1000 hospitalizations)	6.9	5.7	NA	More costly and less effective
**Cost analysis**										
Porgo<br>2019[8]	Level 1 trauma centre;<br>Level II trauma centre,<br>Level III trauma centre,<br>Level IV trauma centre	Direct cost	Median cost per patient	5 885<br>5 885<br>5 885	4 815<br>4 575<br>3 926	NA<br>NA<br>NA	NA<br>NA<br>NA	NA<br>NA<br>NA	NA<br>NA<br>NA	More costly
Mabry<br>2015[9]	Level I and II trauma centres;<br>Level III and IV trauma centres	Direct costs	Median cost per patient	13 712<br>13 712	8 555<br>8 115	NA<br>NA	NA<br>NA	NA<br>NA	NA<br>NA	More costly
Goldfarb<br>1996[10]	Level I trauma centre;<br>Level II trauma centre<br>Non-trauma centre	Direct cost	Adjusted mean charges per patient	12 360<br>12 360	4 660<br>8 237	NA<br>NA	NA<br>NA	NA<br>NA	NA<br>NA	More costly

‡Outcomes are reported without cost conversion
†Only differential effectiveness was calculated
*Only differential costs were calculated
QALY, Quality-Adjusted Life Years
'Ideal' trauma system: it is a trauma system that includes all the components identified with optimal trauma care, such as prevention, access, prehospital care and transportation, acute hospital care, rehabilitation, and research activities
NA: Not Applicable

Online Supplement. Glossary of terms for economic evaluations

Economic-related terms	Definitions
Cost analysis	A process of assessing and quantifying the costs associated with a particular intervention, program, treatment, or service
Cost-consequence analysis	Type of economic evaluation in which costs and outcomes are listed separately in a disaggregated format, without aggregating these results (e.g., usually in incremental cost-effectiveness ratio)
Cost-effectiveness	A type of economic evaluation in which the outcomes are measured in terms of natural (health) units, such as improvement in adherence to recommended care or clinical event avoided
Cost-utility analysis	A type of economic evaluation in which the outcomes are measured in utility units, such as in terms of quality-adjusted life-years (QALYs)
Economic evaluation	An analysis comparing two or more interventions in terms of both their costs and consequences
Incremental cost-effectiveness ratio (ICER)	Ratio of difference in costs of intervention and alternative to difference in outcomes
Int.$	Cost expressed in international dollars
Model-based study	Mathematical representation of reality that can be used to estimate the cost-effectiveness of health interventions
Quality-adjusted life-year (QALY)	Measure of health outcome that combines effect of intervention on length of life and quality of life
Discount rate	Rate by which costs and benefits occurring in future (typically beyond 1 year) are converted to equivalent present values
Perspective	Viewpoint from which economic analysis is conducted (eg, public payer, society, individual); define which costs will be examined
Time horizon	Period of time over which costs and outcomes are measured in economic evaluation
Willingness-to-pay threshold	Maximum amount of money an organization is willing to pay for particular outcome or benefit
Source: Economic evaluations of audit and feedback interventions: a systematic review.	

References

1. MacKenzie EJ, Weir S, Rivara FP, et al. The value of trauma centre care. J Trauma. Jul 2010;69(1):1-10.
2. Durham R, Pracht E, Orban B, Lottenburg L, Tepas J, Flint L. Evaluation of a mature trauma system. Ann Surg. Jun 2006;243(6):775-783; discussion 783-775.
3. Séguin J, Garber BG, Coyle D, Hébert PC. An economic evaluation of trauma care in a Canadian lead trauma hospital. J Trauma. Sep 1999;47(3 Suppl):S99-103.
4. O'Kelly TJ, Westaby S. Trauma centres and the efficient use of financial resources. Br J Surg. Oct 1990;77(10):1142-1144.
5. Bauman ZM, Cemaj S, Patel N, et al. "Peas in a Pod": Clustering minorly injured trauma patients together during their hospitalization results in decreased hospital costs and fewer inpatient complications. Am J Surg. 2022/07// 2022;224(1 Pt A):106-110.
6. Scott M, Abouelela W, Blitzer DN, Murphy T, Peck G, Lissauer M. Trauma Service Utilization Increases Cost But Does Not Add Value for Minimally Injured Patients. Value Health. Jun 2020;23(6):705-709.
7. Zocchi MS, Hsia RY, Carr BG, Sarani B, Pines JM. Comparison of Mortality and Costs at Trauma and Nontrauma Centres for Minor and Moderately Severe Injuries in California. Ann Emerg Med. Jan 2016;67(1):56-67.e55.
8. Porgo TV, Moore L, Truchon C, et al. Patient-level resource use for injury admissions in Canada: A multicentre retrospective cohort study. Injury. Jun 2019;50(6):1192-1201.
9. Mabry CD, Kalkwarf KJ, Betzold RD, et al. Determining the hospital trauma financial impact in a statewide trauma system. J Am Coll Surg. Apr 2015;220(4):446-458.
10. Goldfarb MG, Bazzoli GJ, Coffey RM. Trauma systems and the costs of trauma care. Health Serv Res. Apr 1996;31(1):71-95.
